# Vaccine Development for Influenza Virus

**DOI:** 10.3390/vaccines13101068

**Published:** 2025-10-19

**Authors:** Ganesh Yadagiri, Mingtao Zeng

**Affiliations:** 1 Department of Pharmacology and Toxicology, National Institute of Pharmaceutical Education and Research (NIPER), Kolkata 700054, India; 2 Center of Emphasis in Infectious Diseases, Department of Molecular and Translational Medicine, Paul L. Foster School of Medicine, Texas Tech University Health Sciences Center El Paso, El Paso, TX 79905, USA

## 1. Background

Influenza is a contagious respiratory viral infection that remains a persistent global public-health threat responsible for three to five million severe illnesses and 290,000 to 650,000 respiratory deaths annually. Vaccination remains the strategy effective strategy for preventing influenza virus infections and limiting its transmission. However, the influenza virus undergoes extensive genetic variability through antigenic drift and antigenic shift, leading to the emergence of new seasonal or pandemic strains that can escape pre-existing population immunity. This genetic flexibility only reinforces the need for novel and flexible vaccine platforms.

In recent years, significant advancements in vaccine technologies, including mRNA platforms, recombinant proteins, advancements in adjuvant research, and nanotechnology-based delivery systems, have the potential to significantly change the prevention of influenza virus infection. Recognizing this momentum, the Special Issue “Vaccine Development for Influenza Virus” was launched to bring together cutting-edge research and comprehensive reviews addressing immunological mechanisms, technological innovation, and translational challenges in the development of next-generation influenza vaccines.

## 2. Collection of Special Issue Articles

In this Special Issue, entitled “Vaccine Development for Influenza Virus”, a total of nine papers were published—including eight original research articles and one comprehensive review—highlighting significant advancement in the field of influenza vaccine development.

Epitope Variation in Hemagglutinin and Antibody Responses to Successive A/Victoria A(H1N1) Strains in Young and Older Adults Following Seasonal Influenza Vaccination.

The first article published in this Special Issue, authored by Espinar-García et al. (University of Cordoba, Spain), investigates how original antigenic sin (OAS)—also referred to as antigenic imprinting—affects antibody responses to sequential influenza vaccination [1]. They evaluated immune responses against two antigenically similar A/Victoria A(H1N1) strains, (A/Victoria/2570/2019 and A/Victoria/4897/2022), both in young adults and older adults, by stratifying by cytomegalovirus (CMV) serostatus. In young and older adults, the participants had a higher antibody titer against the 2019 strain, despite vaccination against the 2022 strain, indicating a memory bias toward previously encountered epitopes. Furthermore, functional bioinformatic B-cell epitope mapping of antigenic drift indicated subtle amino acid sequence changes in the globular head of hemagglutinin, leading to the immune recall of prior antigenic exposure. This pilot study of OAS elegantly demonstrates the interplay of antigenic drift and immune history to shape the ability of the vaccine to elicit immunity and illustrates the need to develop vaccines that are broader and more adaptable in the context of both age and immune imprinting effects.

The second article in this Special Issue, authored by Paulo Lee Ho et al. (Bio Industrial Center, Butantan Institute and Butantan Foundation, São Paulo, Brazil), focuses on developing pandemic influenza vaccine candidates as preparedness against the emerging highly pathogenic avian influenza (HPAI) H5N1 viruses [2]. Specifically, the authors describe the production and immunogenicity of three split and inactivated egg-based vaccines developed from different clades—A/Astrakhan/3212/2020 (H5N8), A/duck/Vietnam/NCVD-1584/2012 (H5N1), and A/Anhui/1/2005 (H5N1)—and formulated with IB160 (squalene-based oil in water emulsion adjuvant). Two adjuvanted doses elicited robust clade-specific antibody responses with high hemagglutination-inhibition and micro neutralization titers, indicating higher immunogenicity and strengthening the role of adjuvant optimization and prime–boost regimens. Importantly, it was revealed that there is some limited cross-reactivity with available older H5N1 vaccine strains, underscoring the necessity for ongoing antigenic updates and fast-tracked modifications of existing egg-based production systems in scenarios where a pandemic occurs.

In the third article, Hye Eun Lee et al. (SK Bioscience Co., Ltd. and Korea University Ansan Hospital, Republic of Korea) examined the safety and infection rates after vaccination with the cell culture-derived quadrivalent influenza vaccine (SKYCellflu^®^) in children aged 6–35 months during the 2023–2024 influenza season [3]. In a prospective, multi-center surveillance study of 333 subjects, there were no vaccine-related serious adverse events reported, and the overall rate of influenza infection was 4.5%. Notably, those who received two doses of vaccine in the 6–11-month age group had a lower infection rate (0.8%) compared to those who received one dose, which had a rate of 3.8%, supporting the benefit of a two-dose schedule for vaccine-naïve infants. These data support continuing the evaluation of cell culture-based vaccines.

The fourth article by Hai Xu et al. (Jiangsu Academy of Agricultural Sciences, China) presents a dual-targeted fusion protein approach to improving the potency of the inactivated H9N2 avian influenza vaccine [4]. In this study, fusion proteins of Griffithsin (GRFT) with dendritic cell targeting nanobodies (GRFT-VHH54 and GRFT-VHH74) greatly expanded humoral, mucosal, and cellular immune responses in chickens. More importantly, the adjuvanted vaccine with GRFT-VHH74 provided about 90% protection against a heterologous challenge, demonstrating a promising novel dendritic cell targeting vaccine approach for next-generation influenza vaccines.

The fifth article by Yasuko Hatta et al. (FluGen, Inc., Madison, USA) was on the safety of next-generation single-replication intranasal influenza vaccines (M2SR and BM2SR) [5]. Using ferret and mouse models, they have shown these vaccine viruses would not replicate, shed, or transmit following inoculation, to have minimal risk of reassortment with circulating strains, and to be safe in Phase 1 human trials further demonstrating a non-transmitting profile and safe clinical use, bolstering the unique potential of the M2SR/BM2SR platform of next-generation influenza vaccines to induce both robust mucosal and systemic immune responses. Overall, it would appear these vaccines provide the safety characteristics of inactivated formulations, while retaining the immunogenic benefits and properties seen with live vaccines.

In the sixth article of the Special Issue, Peiqing He and colleagues (Xiamen University, China) describe the creation of a chymotrypsin-dependent live-attenuated influenza vaccine offering broad protection against homologous and heterologous strains [6]. By mutating the hemagglutinin cleavage site of A/California/04/2009 (H1N1) to create a virus that can be activated only by chymotrypsin, this group was able to create an attenuated strain (CA04-F) that is 100 times safer than the parental strain in mice. Intranasal immunization with CA04-F showed stronger humoral and mucosal immunity, and complete protection against lethal doses of both H1N1 and H5N1 viruses. This approach is an innovative and novel strategy for developing safe, broad-spectrum, mucosal live-attenuated vaccines to prevent influenza and other respiratory viruses.

This Special Issue’s seventh paper, by Claudio Costantino and colleagues (University of Palermo, Italy), presents mid-term estimates of influenza vaccine effectiveness (VE) against the A(H1N1)pdm09 subtype during the 2023–2024 influenza season in Sicily Utilizing data from the Sicilian RespiVirNet surveillance system, 1230 patients presenting with influenza-like illness were examined and 29.2% tested positive for influenza, primarily A(H1N1)pdm09 (96.2%) [7]. Employing a test-negative case–control design, the adjusted VE was 41.4% overall, 37.9% in children (7 months–14 years), and 52.7% in older adults (≥65 years). These results further substantiate the moderate protective benefit of the current influenza vaccines and reinforce the continuing value of virological surveillance and antigenic characterization in understanding vaccine match and informing public health guidance for seasonal influenza prevention efforts.

The eighth article, by Pere Godoy and colleagues (University of Barcelona), examined 5080 hospitalized patients with influenza in Catalonia from 2010 to 2020 to evaluate the influence of vaccination and early antiviral treatment [8]. The findings demonstrated that seasonal vaccination, where applicable, and antiviral therapy, increases the likelihood of receiving treatment, were associated with significantly less risk of pneumonia among patients with severe influenza illness when administered within 48 h of symptoms. The study emphasizes the importance of early treatment, including vaccination, to help prevent serious complications from influenza.

The last paper and the ninth paper, authored by Shabi Parvez et al. (India), is a review called “Influenza Virus: Global Health Impact, Strategies, Challenges, and Role of Nanotechnology in Influenza Vaccine Development” [9]. The review discusses the increasing global burden of influenza and the issues associated with contemporary vaccines—for instance, strain mismatch, low efficacy in patients, lack of durability, and reduced responses in older patients. They note that there are promising vaccine platforms based on nanotechnology—like liposomal vaccines, polymeric nanoparticles, and virus-like particles—that may facilitate a better antigen delivery system and improve immune responses to vaccination. Furthermore, the authors suggest that mRNA technology could be utilized in the development of universal vaccines for influenza, which was previously a dream for all global vaccine-preventable diseases. The authors suggest a possible future horizon of nanoparticle-based and mRNA-based vaccines

## 3. Discussion

The articles presented in this Special Issue describe important advances in influenza vaccine research, focusing on innovative vaccine platforms, the science of adjuvants, and delivery systems. Newer methodologies for vaccine platforms such as mRNA, viral vectors, recombinant proteins, and nanocarrier-based vaccines provide vaccine development opportunities to accelerate responsiveness to new influenza strains. Several articles show that mucosal and nasal vaccination is promising to induce durable local and systemic immunity and cross-protection against variant viruses. There are also great advancements in our ability to design more effective vaccines through the omics-based selection of antigens and adjuvants. Together, these studies help to characterize the expanding global efforts for safer, broader, next-generation influenza vaccines that produce long-lasting and universal protection against influenza viruses.

Collectively, these studies emphasize that a universal influenza vaccine—once a distant goal—is now within reach through interdisciplinary collaboration spanning virology, nanotechnology, computational biology, and clinical immunology.

## 4. Conclusions

The investigation in this Special Issue presents exciting developments with respect to the design and development of next-generation influenza vaccines. Advances in mRNA and recombinant platforms, nanotechnology-based delivery systems, and new adjuvant formulations are enabling protective vaccines with greater strength, breadth, and longevity. In addition, new mucosal and intranasal approaches create new possibilities to prevent infection at the portal of entry. It is clear from these studies that global scientific collaboration and technological innovation are closing the gap on the goal of effectively counteracting seasonal and pandemic threats with a universal influenza vaccine.

As Guest Editors, we express our sincere gratitude to all authors, reviewers, and the Vaccines editorial team for their dedication in making this Special Issue a success. We hope these contributions inspire further innovation in influenza vaccine research and development of vaccines for other diseases at large.
